# Radicular Cysts and Periapical Granulomas: Data Documentation for 696 Cases and Findings on Fibrosis, and *Porphyromonas gingivalis* and *Fusobacterium nucleatum* in These Lesions

**DOI:** 10.1002/cre2.70098

**Published:** 2025-02-18

**Authors:** Sirke Virkkunen, Terhi Kaarela, Merja Laine, Auli Suominen, Jaana Hagström, Timo Sorsa, Caj Haglund

**Affiliations:** ^1^ Department of Pathology University of Helsinki and Helsinki University Hospital Helsinki Finland; ^2^ Department of Dentistry University of Turku Turku Finland; ^3^ Department of Oral Pathology and Radiology University of Turku Turku Finland; ^4^ Translational Cancer Medicine Research Program, Faculty of Medicine University of Helsinki Helsinki Finland; ^5^ Department of Oral and Maxillofacial Diseases University of Helsinki and Helsinki University Hospital Helsinki Finland; ^6^ Section of Periodontology and Dental Prevention, Department of Dental Medicine Karolinska Institutet Stockholm Sweden; ^7^ Department of Surgery University of Helsinki and Helsinki University Hospital Helsinki Finland

**Keywords:** apical periodontitis, chronic inflammation, dentigerous cysts, periodontopathogens

## Abstract

**Objective:**

The objective of our study was to re‐evaluate periapical lesions, including radicular cysts (RCs) and periapical granulomas (PGs) for locations, histopathological features, and degree of fibrosis in relation to the inflammatory response. In addition, we examined the presence of *Porphyromonas gingivalis* (Pg) and *Fusobacterium nucleatum* (Fn) since both are widely recognized pathogens in periodontal infections.

**Methods:**

We re‐evaluated samples of RCs and PGs (*n* = 728) and collected data for analyses by IBM's SPSS Statistics. Among these samples, we stained 93 samples to determine the immunoexpression of Pg and Fn. For immunostaining, we used Gingipain R1 antibody for Pg and Rabbit anti‐Fn antibody for Fn.

**Results:**

Fibrosis is associated with mild inflammation. We found a significant positive correlation between Pg and Fn. Thus, these pathogens are likely to occur together in periapical inflammatory lesions. We additionally noted that these periodontopathic pathogens are more likely to be present in RCs than in PGs.

**Conclusions:**

Asymptomatic radiologically diagnosed periapical lesions may not necessarily need root canal retreatment in healthy patients since these lesions may represent scar tissue rather than active apical periodontitis. Clinical and radiological follow‐up is still needed. Yet, periapical lesions, especially cysts, may contain dystopic periodontopathic pathogens, and Pg and Fn often occur together in periapical lesions.

## Summary

- Periodontopathogens *Porphyromonas gingivalis* and *Fusobacterium nucleatum* occur likely together in periapical inflammatory lesions and are more present in cysts than granulomas.
- Inflammation is low within fibrotic lesions: up to one‐fifth of the documented lesions seem to be scar tissue.
- *P. gingivalis* is present in the endothelial cells of the blood vessel walls.

## 1 Introduction

Apical periodontitis is an inflammatory disease of microbial origin, characterized by an inflammatory response and destruction of the periapical tissues (Siqueira and Rôc̦as 2022). It is the most frequent inflammatory lesion of the jaw, with a worldwide prevalence of 52% and the prevalence is higher in developing countries and among individuals with one or more systemic conditions (Tibúrcio‐Machado et al. 2021). Bone destruction is caused by both microbial infection and the immune response as a part of the defense reaction. Depending upon the grade of infection in the root canal, an acute or chronic reaction can occur (Siqueira and Rôc̦as 2022).

Apical periodontitis includes inflammatory radicular cysts (RCs) and periapical granuloma (PG) (Braz‐Silva et al. 2019). Several studies have shown that PG is the most common type of apical periodontitis, with the maxillary anterior region the most affected site (H. P. Lin et al. 2010; Lalonde and Luebke 1968). They are distinguished from one another using the epithelial lining appearing on the walls of RCs. Granulomas consist of granulation tissue with inflammatory cells, fibroblasts, and well‐developed fibrous capsules (Braz‐Silva et al. 2019).

The epithelial lining is most likely derived from the epithelial cell rests of the Malassez as a product of the inflammatory proliferation of the cell rests in the area of apical periodontitis (Wang and Olmo 2022). An RC is originating from PG (Galler et al. 2021; L. M. Lin et al. 2017). The exact mechanism of cyst formation remains unclear.

Both lesions are referred to radiographically as apical periodontitis and are formed when bacteria reach the tooth pulp and cause an infection, leading to pulp necrosis (García et al. 2007). Lesions can only be diagnosed by histological examination. RCs typically present as an osteolytic lesion located in the apex of the teeth upon conventional radiography and via cone beam computed radiography (L. M. Lin et al. 2017).

Some studies suggest that certain radiolucent areas observed in imaging might be related to the healing processes involving the formation of dense collagenous tissue, often referred to as scar tissue. This healing mechanism has been noted in various studies (Nair 2006; Penick 1961; Seltzer et al. 1967). However, our knowledge of how healing occurs after surgical and nonsurgical endodontic treatments remains limited (Nair 2006). Long‐term studies, covering 10–27 years, have tracked asymptomatic cases where persistent thickening of the periodontal ligament showed no significant radiological changes. These findings led to the hypothesis that some changes appearing as apical periodontitis on imaging could represent scar tissue (Molven and Halse 1988; Halse and Molven 2004). However, systematic reviews have found no solid evidence to support conclusions concerning the diagnostic accuracy of a radiological examination to identify scar tissue healing (Petersson et al. 2012).

Root canal infections are polymicrobial, predominantly involving Gram‐negative anaerobic bacteria (Sundqvist 1976; Haapasalo et al. 1986; Sundqvist et al. 1989). Primary infected canals with apical periodontitis differ in species and numbers from secondary infections (Gomes et al. 2004; Tani‐Ishii et al. 1994). Primary infections primarily involve species like *Bacteroides*, *Porphyromonas*, *Prevotella*, *Fusobacterium*, *Treponema*, *Peptostreptococcus*, *Eubacterium*, and *Campylobacter*, while secondary infections show higher prevalence of *Enterococci*, *Streptococci*, *Lactobacilli*, and *Actinomyces*, and fungi such as *Candida* is higher (Neelakantan et al. 2017). *Porphyromonas gingivalis* (Pg), *Fusobacterium nucleatum* (Fn), *Streptococcus salivarius*, *Treponema denticola*, and *Tanerella forsythia* have been found in descending order in symptomatic primary endodontic infections (Zargar et al. 2020). Pg has been identified as a predominant pathogen in all phases of endodontic retreatment and, therefore, related to endodontic treatment failure (Barbosa‐Ribeiro et al. 2021). Fn, frequently found in periapical abscesses (Oguntebi et al. 1982), is a major contributor to endodontic failure and posttreatment apical periodontitis (Prada et al. 2019).

In this study, we aimed to document the data from 728 cases of inflammatory RCs and PGs as well as examine the immunopositivity of Pg and Fn in these lesions. Both these dystopic and proteolytic periodontopathogens are widely recognized as playing a role in the development of periodontal infections together with other oral pathogens (Enersen et al. 2013; Bolstad et al. 1996).

## 2 Materials and Methods

### 2.1 Tissue Samples

Data were collected from the pathology archives of Helsinki University Hospital for patients with diagnosed periapical lesions between the years 2000 and 2013.

**Figure 1:**
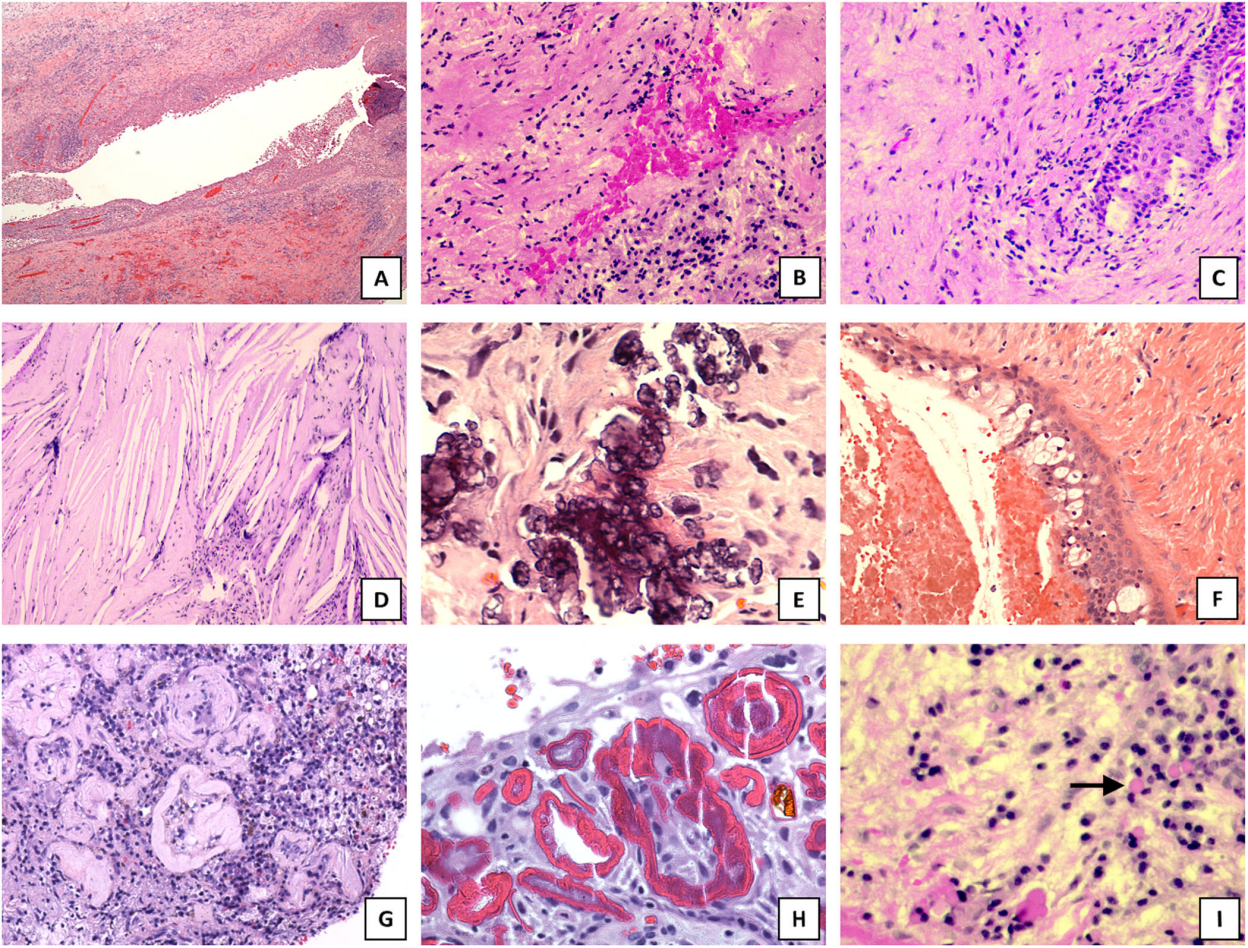
Histological features of periapical lesions. Representative pictures of histological findings in inflammatory periapical lesions in hematoxylin and eosin staining. (A) radicular cyst (magnification ×40), (B) periapical granuloma (magnification ×200), (C) fibrosis in radicular cyst (magnification ×200), (D) cholesterol clefts in radicular cyst (magnification ×100), (E) calcification (magnification ×400), (F) goblet cells in cyst epithelium (magnification ×200), (G) oral pulse granuloma (magnification ×200), (H) Rushton bodies (magnification ×400), (I) Russell bodies (magnification ×400).

In total, we collected 728 samples previously histopathologically diagnosed as RC or PG. We included 696 samples for re‐evaluation by two authors (J.H. and S.V.). Misdiagnosed tissue samples and samples with little tissue material left were excluded from further study. All samples were tabulated based on diagnosis, age, sex, localization, state of inflammation, inflammatory cell types, detection of cholesterol clefts, calcification, respiratory epithelium, goblet cells, foam cells, oral pulse granuloma vacuoles, Rushton bodies, Russel bodies, bacteria plaque, fibrous tissue, and hemorrhage. Histological features are represented in Figure 1. The state of inflammation was scored as follows: 0, fibrotic (as no inflammation cells); 1, mild; 2, moderate; or 3, abundant. Fibrosis was scored as follows: 0, no fibrosis; 1, mild or moderate fibrosis; or 2, moderate or abundant fibrosis. Other variables were tabulated without scoring.

The use of the tissue samples was approved by the Ethics Committee of Helsinki University Hospital (diary number 466/2020), the study protocol was approved by the Helsinki University Research Board, and the samples were provided by the Helsinki Biobank. Due to the retrospective nature of the study, patient consent was not required. This study was conducted with the ethical standards established in the 1964 Declaration of Helsinki and its later amendments.

### 2.2 Statistical Analysis

Data analysis was performed using IBM SPSS Statistics for Windows, version 28.0 (IBM Corp., Armonk, NY, USA). Descriptive statistics, comparative analyses, and Spearman's correlation were calculated. Comparative analyses were performed using the chi‐square test and when assumptions were not valid using the Fisher's exact or likelihood ratio test. We considered a *p*‐value of less than 0.05 as statistically significant.

### 2.3 Immunohistochemistry With Bacterial Antigens

For immunohistochemistry, we collected 100 RC (*n* = 59) and PG (*n* = 34) paraffin‐embedded samples in total representing as wide a range of inflammation levels as possible. Among these, seven samples had too little material left for staining and were thus excluded. For Pg and Fn immunostaining, we used Gingipain R1 antibody for Pg (biorbyt orb243611) since Gingipain represents a specific enzyme for Pg, and Rabbit anti *F. nucleatum* antibody (Diatheva ANT0084) for Fn.

After the slides were deparaffinized and rehydrated, antibodies were applied according to the manufacturer's protocol. The staining was performed using the Envision Flex‐kit (K8000, Agilent Technologies, Singapore). Sections were then counterstained with Mayer's hematoxylin, dried, and mounted before scoring. Negative controls without primary antibodies were included in each staining to confirm the absence of contamination.

### 2.4 Scoring of Immunohistochemistry

The hematoxylin and eosin (HE) glasses were re‐evaluated and immunohistochemical positivities were scored by two researchers (S.V. and J.H.) independently. In cases of disagreement, consensus was reached through discussion. The details of the scoring are summarized in Table 1.

**Table 1:** Details of the antibodies used and scoring.

	Clone	Company	Dilution	Reaction time	Scoring
Gingipain R1 antibody	Polyclonal	biorbyt	1:600	O\N + 5	Low expression, none to mild
					Light expression, moderate to strong
Rabbit anti‐*Fusobacterium nucleatum*	Polyclonal	Diatheva	1:100	O\N + 5	Low expression, none to mild
					High expression, moderate to strong

## 3 Results

The histopathological re‐evaluation in 728 cases is described in Table 2. Thirty‐two cases were excluded due to misdiagnosis or poor tissue samples. Information on the jaw was unavailable in six cases and information regarding the region was unavailable in 36 included cases (Table 2). The most affected sites for RC were the lower molar (24.8%) and upper incisal region (22.0% of cysts). Additionally, the most frequent site for PG was the upper incisal (26.0%) and lower molar (24.0%) region. Lesions were more frequently observed in males than females.

**Table 2:** Frequency (%) of sex, age, localizations, histopathological features, and state of inflammation among 696 cases of periapical inflammatory lesions.

	Total	Radicular cyst	Periapical granuloma
Frequency (%)	*n* = 696 (100.0)	*n* = 541 (77.7)	*n* = 155 (22.3)
Mean age	51.2	51.3	50.9
Sex			
Male	63.2	68.6	43.9
Female	36.8	31.2	56.1
Jaw	*n* = 690	*n* = 536	*n* = 154
Maxilla	49.7	50.9	59.1
Mandible	44.2	49.1	40.9
Region	*n* = 660	*n* = 506	*n* = 154
Incisal	34.7	34.0	37.0
Premolar	21.1	21.7	18.8
Molar	44.2	44.3	44.2
State of inflammation			
Fibrotic	19.3	21.3	12.3
Mild	22.6	22.2	23.9
Moderate	36.1	29.9	57.4
Abundant	22	26.6	6.4
Cholesterol clefts	21.7	26.6	4.5
Calcification	6.6	8.9	0.6
Respiratory epithelium	2.9	3.3	1.3
Goblet cells	1.9	2.4	0.0
Foam cells	14.8	14.0	17.4
Oral pulse granuloma	1.0	1.3	0.0
Rushton bodies	6.0	7.8	0.0
Russel bodies	5.0	2.8	12.9
Bacteria plaque	1.7	2.2	0.0
Hemorrhage	22.6	20.5	29.7

### 3.1 Fibrosis

Fibrosis was detected in 318 of 696 samples included in this study. Table 3 illustrates the relationship between the state of inflammation and the grade of fibrosis. We observed a statistically significant association between fibrosis and low grade of inflammation.

**Table 3:** Crosstabulation between the state of inflammation and fibrosis within 696 samples.

State of inflammation	Fibrosis (%)		
	None	Mild	Abundant
Fibrotic	11 (2.9)	1 (0.8)	122 (61.6)
Mild	55 (14.6)	37 (30.8)	65 (32.8)
Moderate	172 (45.6)	71 (59.2)	9 (4.5)
Abundant	120 (37.0)	11 (9.2)	2 (1.0)
Total	378	120	198

### 3.2 Immunohistochemistry With Bacterial Antigens

**Figure 2:**
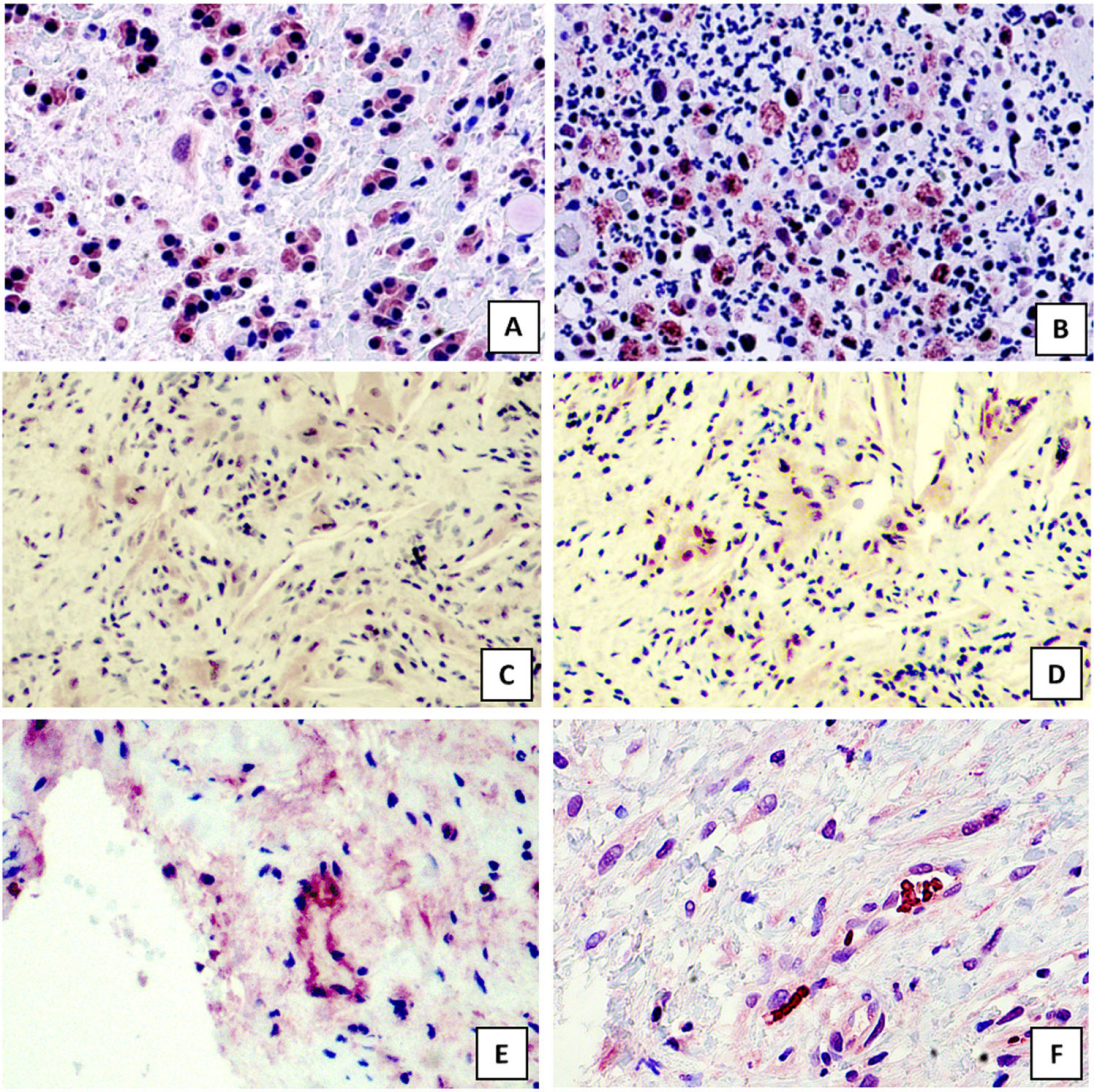
Immunostaining with bacterial antigens. (A and B) Positivity to bacterial antigens in inflammatory cells with Gingipain R1 antibody (A) and Rabbit anti‐*Fusobacterium nucleatum* antibody (B). Magnification ×400. (C and D) Positivity to bacterial antigens in same tissue slides in radicular cyst; Gingipain R1 antibody (C) and Rabbit anti‐*Fusobacterium nucleatum* antibody (D). Magnification ×200. (E and F) Positivity to Gingipain R1 antibody in blood vessel walls of a radicular cyst. Magnification ×400.

In total, 93 samples were stained with the Gingipain R1 antibody and the Rabbit anti‐*F. nucleatum* antibody. Figure 2 illustrates the positivity of staining. Fn was detected in inflammatory cells, sometimes in the epithelium and multinucleated giant cells. Pg was detected in the same cell types and, additionally, sometimes in blood vessel walls. Both were more often present in cysts than in granulomas and often colocated as shown in Tables 4 and 5.

In inflammatory cells, a high expression for Gingipain R1 antibody (GP) was detected in 46 samples and for Rabbit anti‐Fn antibody (FN) in 33 samples. Tables 4 and 5 summarize the specific scoring and features of the samples. We found a statistically significant positive correlation between GP and FN (*p* = 0.003).

**Table 4:** Immunostaining with bacterial antigens in 93 samples.

	Total (*n* = 93)	Radicular cyst (*n* = 59)	Periapical granuloma (*n* = 34)
High in Gingipain R1 antibody (%)	46 (49.5)	33 (71.7)	13 (28.3)
High in Rabbit anti‐*Fusobacterium nucleatum* antibody (%)	33 (35.5)	21 (63.6)	12 (36.4)

*Note:* The samples were stained with the Gingipain R1 antibody for *Porphyromonas gingivalis* and Rabbit anti *Fusobacterium nucleatum* antibody for *Fusobacterium nucleatum*. There was a statistically significant positive correlation between *P. gingivalis* and *F. nucleatum* (*p* = 0.003).

**Table 5:** Crosstabulation for 56 samples high in bacterial antigens at different states of inflammation.

Total high expression in bacterial antigens *n* = 56	Chronic (*n* = 39)	Mixed cell infiltrate (*n* = 9)	Acute (*n* = 3)	Abscess (*n* = 5)
High in Gingipain R1 antibody, *n* = 46	33	8	2	3
% Within the state of inflammation	84.6	88.9	66.7	60
High in rabbit anti‐*Fusobacterium nucleatum* antibody, *n* = 33	20	5	3	5
% Within the state of inflammation	51.3	55.6	100	100

## 4 Discussion

In this study, we collected 728 samples diagnosed as RC and PG, of which 696 samples were included following re‐evaluation. According to the literature, the prevalence of cysts among periapical lesions has shown a wide range of variability in similar study sets (Ramachandran Nair et al. 1996). The high incidence (Table 2) of RCs in our data is likely due to the search methods from the archives: lesions diagnosed as “granulomas” instead of “periapical granulomas” have likely been omitted from the search. In addition, in our experience, clinicians do not routinely send samples of all small lesions attached to apices of the teeth that have been extracted, which may lead to bias in the distribution of the data. A statistically significant majority of the samples were from male patients, possibly indicating that females seek dental treatment more often compared with male patients. Similar results were observed in Oulu among 25‐year‐old persons (Nurminen et al. 2021). Furthermore, two‐thirds of RCs were diagnosed in males, supporting the idea that males seek dental care at a later stage of disease than females since RC evolves from PG (Galler et al. 2021; L. M. Lin et al. 2017). The mean age of patients was 51.2 years.

In the literature, RCs appear to be more common in the anterior region of the maxilla and premolar region of the mandible (Borg et al. 1974). Our study partly contradicts both RCs and PGs, which occurred more often in the upper incisal and lower molar regions. Respiratory epithelium was found in lesions that perforated the maxillary sinus. Supporting our results, Couto et al. (2021) stated in their multicenter study that the molar region was more often the site of these lesions, although no distinction was mentioned between the premolar and molar regions. In addition, Couto et al. (2021) stated that lesions were more often found in the maxilla. This partly contradicts our study since we detected no differences in the location of these lesions in relation to the jaw.

It has been noted that some radiolucent changes can sometimes be due to partial necrosis caused by pulpitis (Motoki et al. 2021) as well as healing through scar tissue (Nair 2006; Penick 1961; Seltzer et al. 1967; Nair et al. 1999). We found a positive significant correlation between fibrosis and low grades of inflammation given that the grade of inflammation was rather low in the fibrotic lesions. As radiologically fibrotic lesions cannot be distinguished from active inflammation, we suggest that some of the asymptomatic radiolucent lesions could be followed up clinically and radiologically if root canal treatment was otherwise successful.

Periodontopathic pathogens were previously isolated from necrotic pulp and apical periodontitis (Rôças et al. 2001; Marinho et al. 2015). We found a significant positive correlation between Pg and Fn, whereby these pathogens are likely to occur together in periapical inflammatory lesions (Enersen et al. 2013; Bolstad et al. 1996). Saito et al. (2008) have demonstrated a synergistic effect between Pg and Fn in periapical periodontitis. In our study, all samples that had Fn contained Pg as well, supporting the suggestion that Pg enhances the growth of Fn. Both Pg and Fn can activate proteolytic enzymes that eventually promote periapical soft and hard tissue destruction by modifying immune responses and activating the host's matrix metalloproteinases (Sorsa et al. 1992; Doron et al. 2014).

In some samples, we observed a positivity for Pg in the endothelial cells of the blood vessel walls as seen in Figure 2. We speculate that these pathogens migrate to the periapical area directly from deep periodontal pockets or via systemic blood circulation. Via blood circulation, these pathogens can migrate to the entire body. In our sample, Pg and Fn were more present in RCs than in PGs. This agrees with our previous study, in which we showed that bacterial lipopolysaccharide (LPS) occurs in Rushton bodies, which are more often detected in RCs than in PGs (Virkkunen et al. 2023). In previous studies, Fn predominated bacterium in abscesses (Oguntebi et al. 1982) and appeared responsible for endodontic failure (Prada et al. 2019); these findings agree with our results regarding the presence of Fn in all acute infection lesions and abscesses, as detailed in Table 5. We speculate that these pathogens eventually can provoke cyst formation. Moreover, we speculate that severe periodontitis makes it possible for these pathogens to enter the apical region. Unfortunately, we did not have data on the periodontal status of these patients.

The strengths of this study lie in the large data set and thorough re‐evaluation of histological samples. Since the material is retrospective, there might be biases in data collection, including incomplete information and variations in diagnostic criteria over time. The difficulties and limitations due to the lack of information regarding any previous symptoms and the periodontal status as well as the reason for extractions in the data set are acknowledged. While this study contributes valuable insights into apical periodontitis, researchers and clinicians should consider these limitations when interpreting and applying the findings since PGs and RCs can be diagnosed only after biopsy and the final diagnosis does not affect the treatment.

## 5 Conclusions

Up to one‐fifth of periapical lesions are fibrotic, whereby a large proportion of the lesions do not contain inflammatory features. This should be borne in mind when assessing the need for further dental treatment and follow‐up.

Periapical lesions, especially cysts, may contain periodontopathic pathogen, and often Pg and Fn occur together, likely provoking more severe inflammation.

## Author Contributions

Conception: all authors. Design: Sirke Virkkunen, Terhi Sorsa, Caj Haglund, Jaana Hagström. Supervision: Caj Haglund, Timo Sorsa, Jaana Hagström. Fundings: Caj Haglund, Timo Sorsa. Materials: Sirke Virkkunen, Jaana Hagström. Data collection and/or processing: Sirke Virkkunen, Auli Suominen, Merja Laine. Analysis and/or interpretation: Sirke Virkkunen, Auli Suominen. Literature review: Sirke Virkkunen, Terhi Kaarela. Writer: Sirke Virkkunen, Terhi Kaarela, Jaana Hagström. Critical review: all authors.

## Conflicts of Interest

The authors declare no conflicts of interest.

## Data Availability

The data that support the findings of this study are available from the corresponding author upon reasonable request.
